# A Continuous Extraction and Pumpless Supercritical CO_2_ Drying System for Laboratory-Scale Aerogel Production

**DOI:** 10.3390/gels2040026

**Published:** 2016-10-01

**Authors:** István Lázár, István Fábián

**Affiliations:** Department of Inorganic and Analytical Chemistry, University of Debrecen, Debrecen H-4010, Hungary; ifabian@science.unideb.hu

**Keywords:** aerogel production, supercritical CO_2_ drying, continuous solvent extraction, liquid CO_2_ extraction, medium temperature process

## Abstract

Production of aerogels starts with solution chemistry and may end with supercritical carbon dioxide drying, which both require a specialized system. Here we present a complete aerogel production system that was developed and used in our laboratory over the last nine years. Our aim was to develop a supercritical dryer and a protocol, whereby the CO_2_ pump can be left out, and the entire flow system is operated by the pressure of the CO_2_ cylinder. Drying pressure and temperature are controlled by the combination of the filling and heating temperatures. A continuous-mode solvent exchange system has also been developed, in which the solvent consumption during the process can be reduced to one-third of the batch method. In the new medium temperature 1.5 L volume supercritical dryer, the temperature is set to a constant 80–82 °C, and the pressure can be in the 90–200 bar range, depending on the conditions. We have performed approximately 200 dryings thus far, and prepared a wide range of monolithic aerogels, from pristine silica aerogels to polysaccharides and collagen. In this paper, we have summarized not only the technical details, but also the work experiences, as well as advantages and disadvantages of the systems.

## 1. Introduction

Aerogels are delicate materials prepared from wet gels by very careful removal of the solvent in a special drying process, which can preserve the original gel structure without collapse. The main reason for collapse is the presence of capillary forces, originating from the surface tension of the liquid filling the gel structure, as well as the adhesion of the liquid to the capillary walls. Under simple atmospheric drying, capillary forces may destroy the original gel structure, thereby leading to significant shrinking and cracking. To avoid structural damages, measures include eliminating the surface tension (supercritical drying); blocking the mobility of the gel and solvent (freeze-drying); or changing the polarities of the capillary walls versus the solvent (ambient drying, or spring-back drying) [1]. Since several modifications of the original processes have been developed so far, their classification may be less straightforward in certain cases.

Freeze-drying is an excellent technique for the removal of water (or some other solvents) from sensitive biological tissues in the frozen state, and is successfully applied to the aerogel production as well [2,3]. Freeze-drying has previously resulted in mostly powder-like materials when used for inorganic aerogels; however, most recently, an improved technique has been published, which can produce monolithic pieces [4].

Ambient (or spring-back) drying is an attractive method of aerogel production since it does not use any complicated instruments and can be scaled up to industrial use [5,6,7,8]. According to the latest developments, small transparent silica monoliths can also be prepared [9]. There is also, a new spray-drying technique to produce powdery aerogels [10].

Supercritical or critical point drying (SCD) is the most frequently applied technique for aerogel production. It is capable of producing not only powdery but large and optically clear monolithic samples, which are used, i.e., in the Cherenkov-counters [11,12,13,14,15,16]. A significant advantage of the SCD processes is that the solvent used in the gelation process may be selected from a very wide range, and the technique is not limited to certain types of gel materials and is rather quite universal. Two basic strategies are the high temperature (HT) and the low temperature (LT) drying [17].

In the HT process, the solvent itself is heated above its critical point after a pre-pressurization step. Since the critical point of the most common organic solvents is above 200 °C, and the critical pressure is in the range of 40–80 bar, supercritical conditions can be reached by heating wet samples covered with the solvent in a sealed autoclave, followed by slow depressurization [18,19,20,21,22]. The process and the instrumentation are simple; it requires no pumps, and may produce aerogels with hydrophobic surface modification directly. Unfortunately, organic solvents represent a fire hazard in case of accidental and uncontrolled release, and the high temperature may damage heat-sensitive materials. A modification of the HT process is when the temperature is kept a bit under the critical temperature of the solvent [23,24].

Supercritical carbon dioxide is used for low temperature drying due to its low critical temperature (31 °C), environmentally friendly nature and non-flammability. In the process, wet gel samples are periodically or continuously flushed with supercritical CO_2_ until all solvents are removed. Regardless of the periodicity, both processes require heat exchangers, and a liquid CO_2_ pump, which is a valued part of the instrument. The continuous LT process may require larger volume of CO_2_, but thorough optimization and modification to an industrial scale can significantly reduce the specific drying costs [25,26]. Under supercritical conditions, some functionalization of the aerogel skeleton can also be achieved [27]. Due to the high scientific and industrial importance of the aerogels, significant efforts were taken to study the effects of drying conditions, diffusion, chemical composition, and drying temperature profiles on the quality of aerogels [28,29,30,31,32,33,34,35].

Our aim was to develop an environment-friendly, solvent-saving, simple and low running-cost system for the preparation of aerogels from gel casting to supercritical drying. An additional requirement was that the construction should be student-friendly, as it was intended for all levels of research, including undergraduate studies. In this paper, we concentrate on the design and work experiences of our gel casting, aging, solvent exchange and medium temperature (MT) pumpless supercritical CO_2_ drying system.

## 2. Results and Discussion

As has been shown in the introduction, liquid organic solvents may be transformed into their supercritical state by simple heating of the drying vessel after certain pre-pressurization with an inert gas. Our concept was to apply that simple approach to liquid carbon dioxide, which by itself provides the necessary pre-pressure that can transform it into the supercritical state by simple heating, without additional steps. The final pressure of the drying chamber is basically determined by the relative volume of the liquid CO_2_ introduced in the chamber, as well as the final temperature. The volume of carbon dioxide intake can be controlled by the initial temperature of the dryer (provided the CO_2_ cylinder is kept at constant room temperature). The required drying pressure is then determined by the final heating temperature of the chamber. Our new process uses the pressure of the carbon dioxide cylinder to provide liquid flows, and is combined with a double temperature control protocol to achieve a wide range of available drying pressures and temperatures. As is shown in the forthcoming sections, this simple method may substitute complicated and costly supercritical pumping systems and provide crack-free monolithic aerogels made of a variety of materials.

The basic technology steps in our aerogel-making protocol are gel casting, aging, solvent exchange, and supercritical drying. Aerogel synthesis in general starts with controlled chemical hydrolysis of precursors, followed by polycondensation of the hydrolysis products, yielding sol particles first, which grow in time and finally form a self-standing gel. Here we give a detailed description of all the steps (not just the drying process) and show some practical hints in order to provide guidelines or a manual for laboratories preparing to get involved in aerogel research, though some of them might be useful for advanced laboratories as well.

### 2.1. Gel Casting, Molds

The first issue is deciding which mold can be used for gel casting. The mold should be chemically resistant, easy to use and, in our case, a cost-effective one. Polymers, like poly(vinyl chloride), polystyrene and polypropylene are prone to stick to the gel too strongly, resulting in damages of the monoliths when removed from the mold. Using commercial silicone spray to cover the surface may help in some cases, but silicone oil residue may build in the surface of the gel, which may be unwanted. Poly(tetrafluoro ethylene) (PTFE) proved to be the most advantageous construction material. Unfortunately, machined PTFE molds with proper enclosures were not available, thus we combined materials to solve the problem. 

#### 2.1.1. Cylindrical Molds

**Figure 1:**
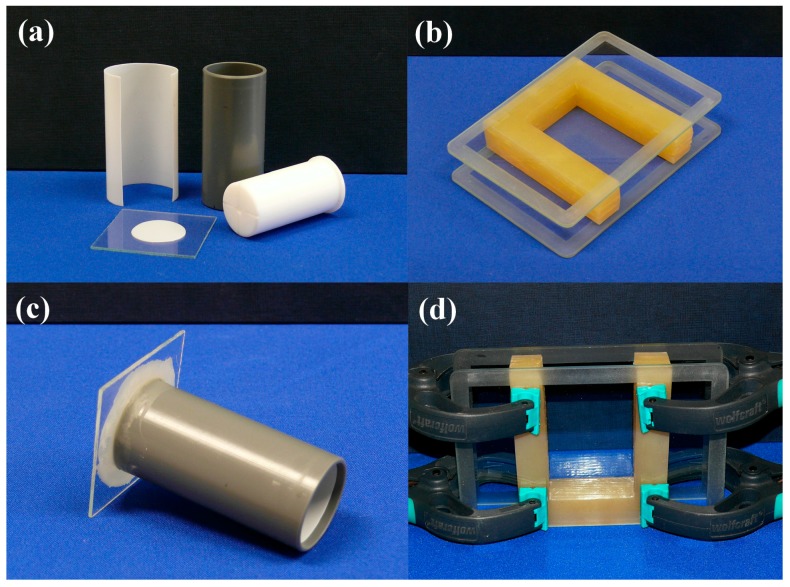
Photographs of disassembled and assembled cylindrical and rectangular molds used for gel casting. (**a**) Parts of cylindrical molds are: machined PVC tubing, PTFE liners, glass base plate, PTFE plunger for extruding alcogel; (**b**) Rectangular mold consisting of a U-shaped mask made of silicone rubber, two glass plates, plus PTFE liners (not shown, for the sake of visibility); (**c**) Assembled cylindrical mold; base plate and tube are glued together with paraffin; (**d**) Assembled rectangular mold with spring clamps (PTFE liner is not shown).

For cylindrical-shaped samples, machined PVC tubes with removable internal PTFE liner is used, which keeps the costs low and provides undamaged gel monoliths (Figure 1a). Pushing-out of the gel from the mold directly to the drying frame is performed very carefully, with the help of a PTFE plunger. It has a coaxial bore and two surface channels to allow passing air into the mold and prevent sticking of the gel to the plunger. The base plate of the mold can be made from the same materials as the mold, but we prefer thin glass plates. Inside the tube, the base surface is covered with a round PTFE liner. The base plate is stuck to the PVC tube by melted paraffin, applied by a brush; paraffin solidifies upon cooling and keeps the parts together with sufficient force to allow moving of molds filled with the reaction mixture or gel. It provides a chemically resistant and easy-to-break seal, which can be removed (break off) with little rotating force, leaving the lower gel surface undamaged (Figure 1c). Due to limited strength of the connection, we do not use that technique for a volume larger than 60 mL. After casting, the molds are sealed tightly with a double layer of a flexible laboratory film (Parafilm) or with aluminum foil, to prevent evaporation. The surface of the gel is covered with a few milliliters of solvent layer (i.e., methanol), when it is aged in the mold.

#### 2.1.2. Rectangular Molds

Rectangular-shaped gels are cast in a custom-made U-shaped silicone template covered on two sides with glass plates (Figure 1b). The internal glass surface is covered with thin PTFE foil stuck to the surface by a high-vacuum silicone grease. The parts are held together by spring clamps (Figure 1d). After casting, the mold is sealed with a rectangular silicone plug to prevent evaporation. The surface of the gel is covered with a few milliliters of solvent layer (i.e., methanol), when it is aged in the mold. Removal of the gel is performed by laying down the set on the table without clamps, rotating and sliding aside the top cover glass plate, followed by stretching out of the U-template. Finally, the gel is transferred to the drying frame by sliding in (this step requires some practice, and the help of a thin PTFE sheet placed on the frame temporarily).

### 2.2. Frames, Sample Holders, Containers

**Figure 2:**
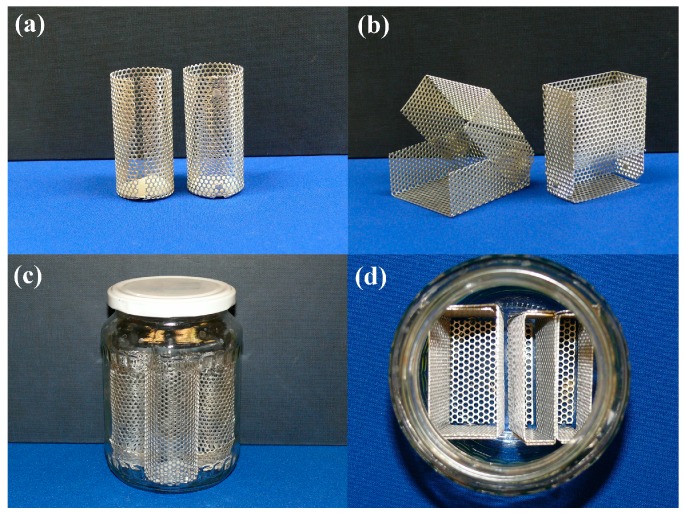
Cylindrical and rectangular frames for holding, aging, solvent exchanging and drying of wet gels, made of punched aluminum plates. All frames are customized, (**a**) and (**b**) free-standing frames, (**c**) and (**d**) frames placed in a fruit jar to show space filling.

Drying frames or shaped sample holders are made of punched aluminum plates of 0.75 mm thickness. They can be made into any shape fairly easily, and their preparation requires very little practice. Cylindrical frames can be assembled with aluminum rivets, but we have found open split structure with a bottom inbound edge covered with filter paper discs quite satisfactory (Figure 2a).

Rectangular frames are designed to be foldable, as the transfer of the monoliths requires more freedom of movements (Figure 2b). The wet gels are transferred, treated and stored in the frames throughout the entire process. Since evaporation of the solvent (that is drying-out of the gel) must be avoided, all aging, chemical treatment and solvent exchange steps are performed in well-closed containers. Instead of using laboratory cylinders with ground joints, we found commercial fruit jars to be reliable and inexpensive containers. The lid fits with a thread hermetically, and the internal surface of the lid is covered with a thin plastic layer. The sizes of the frames are designed to fit the jars, meaning that four cylindrical, two thick or four thin rectangular frames can be placed in a jar at a time (Figure 2c,d). Approximately 350–500 mL of aging mixture or exchanging solvent is filled in the jars, and the samples must be fully covered with liquid all the time.

### 2.3. Aging, Functionalization

Aging may require a longer time to stabilize the gel structure and let the bonds form completely, and sometimes it may need the use of addition reagents [28,36,37,38,39,40,41]. Since it is a very specific process, we will not elaborate on the chemicals used, but refer the reader to the corresponding literature. Aging can be performed either in the original mold (sealed hermetically), or in separate vessels after wet gels are placed in the frames.

Chemical modification, such as hydrophobization, can be performed on the wet gels. For making a hydrophobic silica surface, we use hexamethyl disilazane as a reagent in dry methanol. Trimethyl chlorosilane must be avoided when the gels are in aluminum frames. Hydrochloric acid is liberated in the process, and it corrodes aluminum fairly rapidly. In such cases, a manually punched polypropylene cylinder is used.

### 2.4. Solvent Exchange

Solvent exchange is a critical part of the process, because water content must be removed from the wet gels very carefully. Our supercritical drying process uses acetone as the final solvent, but other organic solvents can be handled as well. Residual water may damage the gel structure upon drying, resulting in opalescent, shrunk and cracked aerogels instead of transparent and crack-free monoliths.

**Figure 3:**
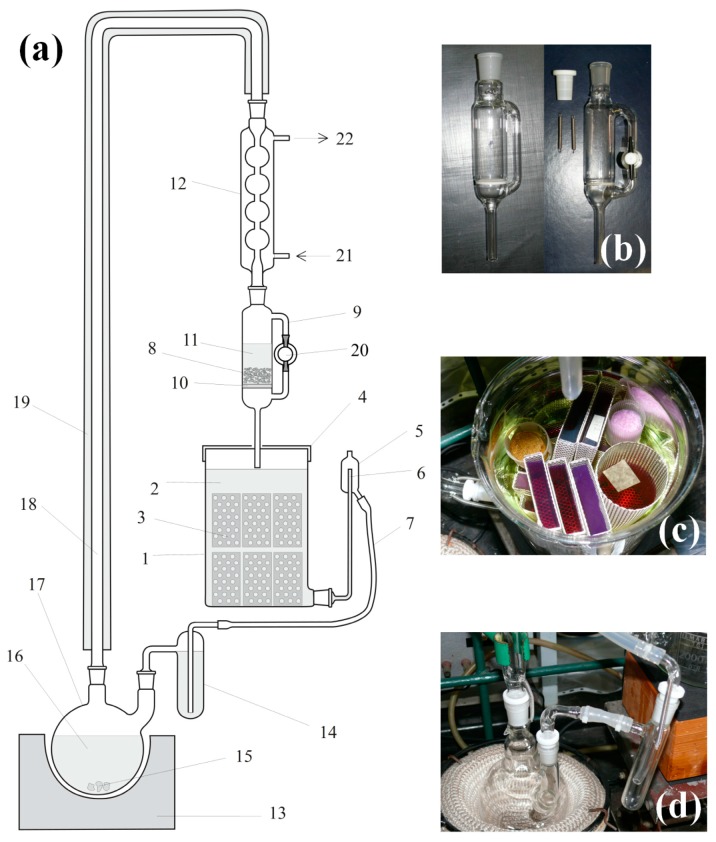
(**a**) Structural drawing of the continuous extractor and solvent regeneration unit, and photographs of (**b**) two types of drying agent holder; (**c**) different kinds of solvogel samples loaded in the extraction chamber; and (**d**) solvent seal. Numbers: (1) extraction chamber, 2 L volume; (2) solvent (acetone); (3) solvogel samples in punched aluminum frames; (4) lid; (5) overflow buffer; (6) solvent level limiter; (7) silicone rubber tubing; (8) anhydrous drying agent Na_2_SO_4_ or MgSO_4_; (9) drying unit with side arm; (10) fritted disc; (11) freshly distilled solvent; (12) condenser; (13) safety electric heater; (14) solvent seal; (15) boiling chips; (16) solvent; (17) two-necked round-bottom flask with ground joints; (18) vapor tube; (19) heat insulation; (20) PTFE stopcock; (21) cooling water in; (22) cooling water out.

In general, the solvent exchange is performed over several steps. Although using a copious amount of fresh and anhydrous exchanging solvents is the easiest way to perform the task, unfortunately, that would result in a copious volume of waste solvent, which should be avoided. We have developed a solvent-saving procedure to reduce solvent consumption. In general, the gelation reaction is performed in a mixture of an organic solvent (typically methanol, ethanol, isopropanol) and water, plus catalyst. After aging and modifications, we soak the gels in a mixture of the same solvent plus acetone, increasing the acetone content in 25% steps to 100%, then soak the gels in pure acetone; each step lasts at least one day or more. After that we transfer the gels in the solvent regeneration extractor, and distill fresh acetone over them for at least three days (Figure 3a).

### 2.5. Continuous Solvent Regeneration and Extraction Unit

Structural drawing of the continuous-mode solvent regeneration and extraction unit is in Figure 3. The main units of the extractor are the distillation flask (Figure 3d), the drying unit (Figure 3b), and the extraction chamber (Figure 3c). Detailed description of the extraction process can be found in Appendix A. 

The extraction chamber can be used for adsorption of different materials, like indicators, in the gels. In Figure 3c, all violet- and red-colored gels were stained by treating them with crystal violet, and then the excess was extracted with acetone. When the solvent exchange/extraction is complete, gels are stored in closed fruit jars under dry acetone.

Special attention should be paid to the proper sample handling in the extractor chamber in order to avoid possible cross-contaminations. As a general rule, gel samples containing functionalizing agents, surface modification reagents, or highly soluble materials like fluorescent dyes should be handled separately. Only gels of identical types should be extracted in the same solution.

### 2.6. Pumpless Supercritical CO_2_ Dryer

#### 2.6.1. Dryer Construction

**Figure 4:**
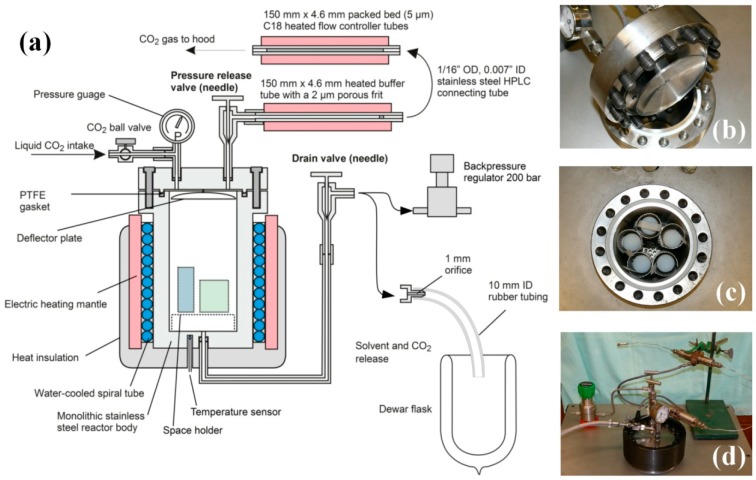
(**a**) Functional drawing of the supercritical dryer, and photographs of (**b**) unassembled reactor lid with deflector plate; (**c**) reactor body containing freshly dried cylindrical aerogel monoliths, and (**d**) assembled reactor with flow controller tubes and back pressure regulator unit.

We have designed a supercritical dryer, which does not require the use of a liquid CO_2_ pump, and the system is operated by the original pressure of the CO_2_ cylinder, plus the pressure increase of carbon dioxide occurring in the liquid to supercritical transition. The cross-sectional drawing of the system is in Figure 4a. Panels b–d in Figure 4 shows critical parts of the dryer: (b) the lid with male-type joint, pressure gauge and deflector plate; (c) the reactor body with a female-type joint with PTFE gasket, as well as freshly dried cylindrical aerogel monoliths, and (d) the assembled dryer unit with safety lock ring (dark), heated flow-controller tubes and back pressure regulator. More technical details can be found in Appendix B. 

#### 2.6.2. Liquid CO_2_ Extraction and Supercritical Drying

Supercritical drying of wet gels is combined with a liquid CO_2_ extraction of acetone. Solubility of acetone in liquid carbon dioxide is quite limited, thus in the first step, acetone is replaced with liquid CO_2_ and drained. In the second step, acetone residue is washed out with liquid CO_2_. In the third step, the reactor is sealed, heated to approx. 80 °C, equilibrated for 3 h, then depressurized at a rate of approx. 1–2 bar/min. 

**Figure 5:**
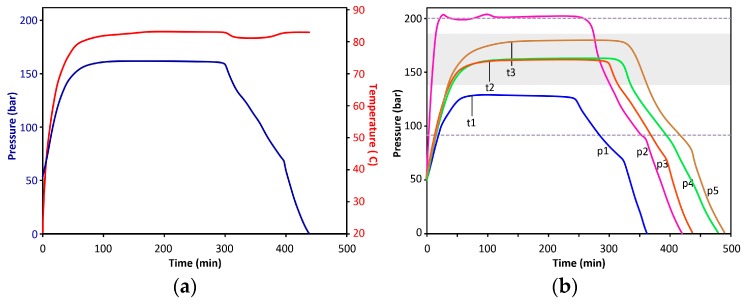
(**a**) Pressure and internal temperature diagrams of a typical supercritical drying process. Moderate internal temperature drop can be observed in the supercritical region of decompression; (**b**) Pressure diagrams of characteristic MT supercritical dryings. Grey area indicates preferred drying conditions, dashed lines show the lowest and highest pressure under which successful dryings were performed. (p1) Rapid drying with low gel load; (p2) Overcharged reactor, CO_2_ was released as the pressure reached the upper limit; (p3–p5) Characteristic MT SC dryings with full gel loads.

The technical steps of the extraction and drying process are summarized in Figure S1 and described in Appendix C. A typical internal temperature and pressure diagram can be seen in Figure 5a. Other characteristic pressure diagrams, including examples of undercharged and overcharged reactors, are shown in Figure 5b. The time necessary to reach pressure stabilization (t1–t3) depends on the starting volume of LC-CO_2_ in the reactor, as well as, to a lesser extent, the number of aerogel monoliths.

A linear depressurization curve of the chamber would be ideal to provide the lowest level of mechanical stress to the aerogel monoliths. However, it can be hardly achieved by simple manual control. The depressurization process may be calculated/modelled by using the van der Waals equation as shown in Appendix D.

**Figure 6:**
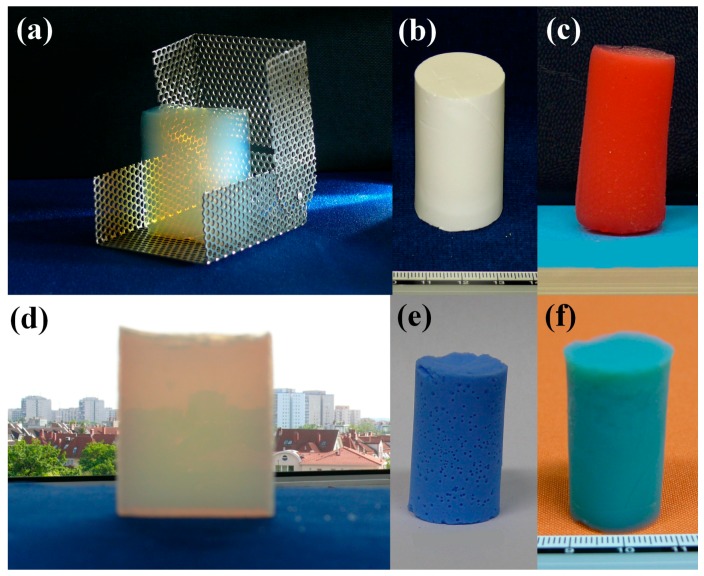
Representative samples of aerogels prepared by the process described in the text. (**a**) Acid-base catalyzed silica aerogel monolith placed in the drying frame for size comparison; (**b**) Silica aerogel-based artificial bone substitute material; (**c**) Fluorescent silica aerogel stained with Safranin T; (**d**) High-strength semi-transparent collagen-silica hybrid aerogel; (**e**) Silica aerogel covalently functionalized with a macrocyclic copper complex; (**f**) Chromia-silica hybrid aerogel.

So far, a large number of aerogel samples have been synthesized by the method described above. Not only silica aerogels, but their covalently functionalized derivatives, organic and inorganic hybrids and composites, iron oxohydroxide, alumina and tin(IV) oxide gels were also prepared. A few examples of monolithic aerogels can be seen in Figure 6.

## 3. Conclusions

The sample preparation technique, from gel casting to solvent exchange, presented here represents a clear and simple way to produce wet gel samples for supercritical drying. Most of the components can be found in an average chemical laboratory, or can be manufactured by a glass-blowing artist, or even made manually.

### 3.1. Continuous Extraction and Solvent Exchange

The continuous extractor provides significant solvent volume reduction in the process, thus reducing material costs and toxic waste management fees. The work principle is clear; it combines a continuous solvent recirculation, regeneration and drying unit with continuous extraction of the wet gels. Furthermore, it gives the possibility of chemical modification of the wet gels in the extraction chamber, followed by direct and complete washing out of the reactants with freshly distilled solvent. The unit can be used for the most commonly used solvents, and the construction allows the instrument to be scaled up (or down), if necessary. A smaller 200 mL unit is also in use for the extraction and solvent exchange of individual samples, which cannot be kept in the large unit, due to leaking of either chemical functionalizing agents or fluorescent dyes.

### 3.2. Comparison of High, Low and Medium Temperature Drying Processes

The principle we use in the MT supercritical dryer is a hybrid of two basic drying strategies, namely the HT and LT approaches, combined with a liquid CO_2_ extraction/solvent exchange step. We use supercritical carbon dioxide drying like in the LT techniques. However, SC-CO_2_ is generated in situ from liquid CO_2_, as in the HT process, where the solvent is heated up above its critical temperature. The medium temperature process (MT) we use (80 °C) applies higher temperature than the LT processes (40 °C), but is much lower than that used in the HT processes (above 200 °C). The basic difference from LT techniques is that our MT process does not require the use of a costly cooled CO_2_ pump. The system is operated by the pressure of the CO_2_ cylinder, and the supercritical medium is generated from liquid CO_2_ directly in the dryer. As a prerequisite for successful operation, all samples should be free of any other solvents, which could increase the critical point, or would form a low-solubility liquid phase within the gels. Removal of solvents is achieved by a semi-continuous liquid CO_2_ extraction before the supercritical transition, and it is performed also in the dryer unit.

The net full processing time, including LC-CO_2_ extraction and supercritical drying, is approximately 16–18 h. The length of supercritical drying only is 6–8 h. Liquid CO_2_ consumption is 4–5 kg/batch. (One batch may contain ten cylinders of 28 mm OD × 50–60 mm L dimensions, or four prisms of 2.2 × 5 × 6 cm^3^_,_ or two prisms of 2.2 × 5 × 6 cm^3^ plus four prisms of 1.1 × 5 × 6 cm^3^ dimensions, or twelve prisms of 1.1 × 5 × 6 cm^3^ dimensions). 

### 3.3. Characteristics and Long Time User’s Experiences with the MT Supercritical Dryer

The pumpless MT supercritical dryer has been in operation in our laboratory for nine years, and around 200 dryings have been performed thus far.

The most significant advantage of the construction is that it requires no CO_2_ pump and heat exchangers, significantly reducing the investment cost. All flow operations are powered by the pressure of the carbon dioxide cylinder. It has a very simple, seamless and sturdy construction. Only a one-phase 240 V AC (2 kW) power line and communal cold running water supply are required. The rolling frame allows high mobility and ease of transportation. Only the main PTFE gasket has to be replaced regularly (after approximately 10–15 dryings); other gaskets do not need replacement for years. Due to ease of handling, there is no need for high level technical training. The dryer has been successfully operated by undergraduate and graduate students for the last eight years.

We have also found a few disadvantages of the system. The most important drawback is that it is controlled manually and requires continuous (but not permanent) personal supervision, generally two work shifts per drying. Although heating is automatic, decompression is manually controlled, thus smooth operation depends on the operator’s skills. Efficacy of filling the reactor with liquid CO_2_ (and thus the final pressure) depends on the temperatures of cooling water and the temperature of the CO_2_ bottle, although the process works fine in a wide range of conditions. Needle valves are prone to over tightening and require regular maintenance.

## 4. Materials and Methods

### 4.1. Materials

All materials were of reagent grade except as noted and used without any purification. Ammonia solution (25%), technical grade methanol and acetone was purchased from Molar Chemicals Kft. (Halásztelek, Hungary). Argon gas cylinder (4.6) and carbon dioxide cylinder equipped with dip tube (Carbogen) were provided by Linde Gáz Magyarország Zrt (Budapest, Hungary). Silane reagents and anhydrous magnesium sulfate were purchased from Sigma-Aldrich (Budapest, Hungary). Steel pressure tubings, pressure gauge, connecting elements, needle valves, and back pressure regulator were purchased from Swagelok and Linde Gáz Magyarország Zrt. (Budapest, Hungary).

### 4.2. Instruments

Parts of the continuous glass extractors were custom made by András Donka in the glass-blower workshop of the Department of Inorganic and Analytical Chemistry, University of Debrecen, Debrecen, Hungary.

The supercritical dryer was manufactured and assembled in the workshop of the Nuclear Research Institute of Hungarian Academy of Science (Atomki, Debrecen, Hungary). Rolling frame, support unit, heating and cooling system were custom made by Goodwill Trade Kft. (Hajdúböszörmény, Hungary).

### 4.3. Synthesis of Silica Aerogels by Acid-Base Catalyzed Process (Samples in Figure 6a,f)

The synthesis is based on a general acid-base catalyzed process widely known in the literature. However, we use boiling temperature in the acid hydrolysis step to reduce the necessary time. Reaction conditions and reagent concentrations are set to reach the gelation point within 8–15 min. Two solutions (A and B) were prepared. Solution A contained tetraethyl orthosilicate (TEOS) (10.00 mL, 44.78 mmol) and aqueous hydrochloric acid solution (1000 µL, 0.20 M) in ethanol (44.0 mL). Solution B contained aqueous ammonia solution (1500 µL, 25% m/m) and ethanol (16.0 mL). Solution A was heated to boiling and refluxed for 5.0 min, then cooled down to room temperature in a cold water bath. The solution was transferred into a 100 mL Erlenmeyer flask, magnetically stirred, and solution B was added under vigorous stirring. The reaction mixture was poured in a rectangular mold and let to gel and age in the mold for one day. The surface was covered with a layer of ethanol. After aging, the gel was transferred into a drying frame and soaked first in ethanol, then in increasing acetone level ethanol until 100% acetone was reached. After three days in acetone, the sample was placed in the continuous extractor and dried and extracted with acetone for three days. The sample was put in the supercritical dryer and dried according to the protocol described in Appendix C. Pristine aerogel received after SCD was heated in a furnace at 500 °C for 8 h to reach final strength; the 1.8 cm thick aerogel monolith can hold 10 kg weight easily, without cracking (see Figure S2).

## Supplementary Materials

The following are available online at http://www.mdpi.com/2310-2861/2/4/26/s1, Figure S1: Visualization of important operation steps and valve settings of liquid carbon dioxide extraction and medium temperature (MT) supercritical drying of aerogels. Red circles: valve closed, green circles: valve open, Figure S2: Acid-base catalyzed aerogel sample holding 10 kg weight without cracking, Figure S3: Change of pressure (panel left) and pressure drop (panel right) as a function of time, calculated by the van der Waals equation for modeling a constant volumetric rate of 1.3 L/min (STP) decompression process of a 1.5 L dryer, which was filled with 1 L of liquid carbon dioxide at 291 K temperature and 5.4 MPa pressure, and heated to 353 K before decompression, Video S1: Evaporation of a dry ice sample containing acetone, Video S2: Evaporation of a dry ice sample free of acetone.

## Abbreviations

The following abbreviations are used in this manuscript:

HT	high temperature
LC-CO_2_	liquid carbon dioxide
LT	low temperature
MT	medium temperature
PTFE	poly(tetrafluoro ethylene)
PVC	poly(vinyl chloride)
SC-CO_2_	supercritical carbon dioxide
SCD	supercritical drying
TEOS	tetraethyl orthosilicate
TMOS	tetramethyl orthosilicate

## Appendix A

### Continuous Solvent Extractor—Work Principles, Operation

In the following description, the solvent is limited to acetone, but the apparatus can be used for any solvents whose boiling point is low enough to let the vapor pass through the vertical vapor tube and enter the condenser.

When the instrument is operated, acetone in the distillation flask, and the drying agent anhydrous MgSO_4_ or Na_2_SO_4_ in the drying unit must be changed daily. Two types of drying unit can be used. The simple unit does not contain a stopcock; it has only a side arm, which serves as a vent/overpressure protection and solvent overflow release in case the drying agent would be blocked (Figure 3b left panel). The advantage of this construction is its simplicity; the disadvantage is that it takes a long time to dry out after washing. A more complicated unit with a PTFE stopcock is more advantageous, because the fritted disc can be dried more rapidly by sucking air through it when the stopcock is closed (Figure 3b right panel). A slight disadvantage is that the stopcock is easily left closed when the extractor is reassembled.

The extraction chamber has a level controller side arm, through which acetone flows back to the distillation flask. Acetone flows through the solvent seal, which prevents solvent vapor escape through silicone tubing, and also serves as a secondary overpressure protection in case the drying unit would be jammed when the stopcock is closed. Accidental/sudden solvent flush is confined in the overflow buffer space.

In continuous mode, approximately 4 L of acetone is regenerated and distilled over the samples every hour. Comparing with the simple soaking technique, this process clearly provides a very significant solvent volume reduction. After three days of extraction, not only water but also other soluble contaminants are extracted.

## Appendix B

### Supercritical Dryer Design Considerations

The drying reactor was designed to be extremely safe and sturdy, as it was also intended for undergraduate research; welding was thus not used for the fabrication process. All connecting parts are rated to at least 300 bar (30 MPa) pressure. The reactor body is machined from a single piece of a stainless steel. The wall thickness is at least 20 mm or more. The lid is a 32 mm thick stainless steel plate, through which 16 screws of M12 size ensure safe and stable closure to the body. To protect the operators from a gas burst or flying parts that may occur upon accidental gasket failure or screw breakage, a safety lock ring is applied around the flanges, partially covering screw heads. Heating of the reactor is performed with an electric heating mantle controlled by a digital PID temperature controller. The heating mantle and the reactor body are separated by a coiled steel cooling spiral connected to the laboratory faucet. This water-filled spiral tube acts both as a heat-transfer unit in the warming-up and heating period, and as a cooler after drying was complete. Also, it can protect the reactor from accidental overheating in case of controller failure, as water boils out from the tubes at 100 °C and the empty tubes may act as a heat barrier. Of course, the spiral can be connected to a refrigerating recirculator or laboratory thermostat, if available. Use of a thermostat with antifreeze may extend the temperature range.

## Appendix C

### Loading, CO_2_ Extraction and Drying Protocol

In order to see opened (green) or closed (red) states of valves at different stages of the process, please find Figure S1 in the Supplementary Materials section. Wet gels are loaded in the dryer under 1000 mL of acetone, then the reactor body is sealed and connected to the CO_2_ bottle. First, a very slight overpressure of CO_2_ is applied, and acetone fill is purged out through the drain valve. It is a good practice to use a polypropylene graduated cylinder to measure the effluent volume. It must be in the 995–1000 mL range. Close the drain valve when gas is coming out only.

The ball valve is then opened very slowly, and the reactor is filled with liquid CO_2_ and then remains permanently connected to the bottle. At room temperature, the density of acetone is higher than that of carbon dioxide, thus acetone settles from the gel bodies while being replaced with liquid CO_2_. In this stage the temperature must not drop below 18 °C, because the density of liquid CO_2_ changes rapidly with the temperature, and the order of phases would be inverted, making phase separation impossible. After approximately 30 min, acetone collected under the space holder is released through the bottom drain valve. This step is repeated twice. After that a very slow flow of liquid carbon dioxide is passed through the reactor periodically, while the quality of dry ice collected in the Dewar is checked for the presence of acetone. Two video records are presented in the Supplementary Materials (Video S1 and Video S2), showing residual acetone droplets on evaporation of a dry ice sample (Video S1) and the sublimation of a clean dry ice sample after acetone is washed out completely (Video S2).

When ready, open cooling water to cool down the reactor body to 14–19 °C, depending on the temperature of the running water. Allow 30–60 min time at that temperature in order to cool completely and for as much liquid carbon dioxide as possible to be sucked out of the reactor. After that, all valves are closed and the reactor is heated up to 80–82 °C and kept at that temperature for the entire process. The maximum pressure in the reactor is generally in the 110–170 bar (11–17 MPa) range, depending on the number of samples loaded in it, as well as the cooling temperature and time and the temperature of the room where the CO_2_ cylinder is stored. A back pressure regulator set to approximately 20 MPa may be used if available, but very careful manual pressure control is possible during the heating-up period, if necessary. We have tested the system up to 250 bar pressure, and operate it below 200 bar.

Please be sure that the spiral tube is filled with water, and water is not running! Wait until pressure is stabilized (it requires approximately 90–120 min), then hold it for 2–3 h. After that, assemble both flow controller tubes to the pressure release valve, heat them up to approx. 100–120 °C and start depressurization by the use of the pressure release valve. Rate of depressurization should be kept in the 1–2 bar/min range. The higher the rate of depressurization, the higher the probability of crack formation. Stronger gels may remain undamaged even at 4 bar/min; sensitive aerogels may be cracked at 1 bar/min.

When the internal pressure has dropped below critical pressure, the second flow controller tube is removed, and pressure release is continued. Depending on the initial pressure, depressurization takes approximately 2–4 h. When pressure has dropped to zero, the flow controller tube is removed, argon gas is introduced through the pressure release valve (opened full), and the internal space is flushed through the drain valve (opened fully) to remove carbon dioxide as well as acetone vapor residues. After that, heating is turned off, the reactor is cooled down with running water. When all connected tubes are removed, the reactor is opened and all aerogels are ready to be removed.

## Appendix D

### Calculation/Modelling of the Depressurization Curve

The change of pressure during the depressurization process may be computed/modelled by the van der Waals’ equation (Equation (D1)), using the parameters *a* = 0.364 Pa·m^6^·mol^−2^ and *b* = 4.267 × 10^−5^ m^3^·mol^−1^.

(D1) $$\left(p+\frac{n^{2}a}{V^{2}}\right)\left(V-nb\right)=nRT$$

where *p* is the pressure, *T* is the temperature, *V* is the dryer’s volume, *n* is the amount of CO_2_ (mol number), *a* and *b* are the van der Waals parameters.

Initial conditions used for modelling of an actual drying process were: 1.5 L chamber volume, filled with 1.01 L of liquid carbon dioxide at 291 K temperature and 5.40 MPa pressure. In the reactor, the calculated amount of carbon dioxide is 18.11 mol. Final drying temperature of the reactor is 353 K. In the modelling process, a constant gas take-off rate of 1.3 L/min (STP) was used for the calculations. Under the given conditions, a total time of 341 min was necessary to reach the standard room pressure. The remaining chamber pressure, as well as the rate of pressure change (pressure drop) were calculated for each 0.1 mole fraction segment, and plotted against time (Figure S3).

The results showed an S-shaped curve, indicating that a simple constant mass/volume control of the outgoing gas would provide an uneven pressure change in the dryer. This calculation is in very good accordance with our observations. In the first period of decompression, the system is very sensitive to the release rate, but the sensitivity drops significantly by the middle of the process. An initial 1.04 bar/min depressurization rate goes down to 0.31 bar/min value in the middle of the curve, then returns to 0.94 bar/min by the end of the process.

The practical consequence is that the researcher must pay special attention when controlling the depressurization process to avoid sudden pressure drops. Mechanical stress from a sudden pressure drop may initiate cracking. In addition, a sudden adiabatic cooling of the internal space occurs, which may lead to transient condensation of the medium when the system is used near the actual critical point. Condensation would result in opalescent aerogels, shrinking and formation of cracks.

## References

[1] Aegerter M.A., Leventis N., Koebel M.M. (2011). Aerogels Handbook.

[2] Chen Y., Ng K.C., Yan W., Tang Y., Cheng W. (2011). Ultraflexible Plasmonic Nanocomposite Aerogel. RSC Adv..

[3] Orsolya Czakkel O., Marthi K., Geissler E., László K. (2005). Influence of Drying on the Morphology of Resorcinol–Formaldehyde-Based Carbon Gels. Microporous Mesoporous Mater..

[4] Pons A., Casas L., Estop E., Molins E., Harris K.D.M., Xu M. (2012). A New Route to Aerogels: Monolithic Silica Cryogels. J. Non-Cryst. Solids.

[5] Schwertfeger F., Frank D., Schmidt M. (1998). Hydrophobic Waterglass Based Aerogels without Solvent Exchange or Supercritical Drying. J. Non-Cryst. Solids.

[6] Durães L., Matias T., Patrício R., Portugal A. (2013). Silica Based Aerogel-like Materials Obtained by Quick Microwave Drying. Mater. Werkst..

[7] Guo Y., Wang H., Zeng L. (2015). SiO_2_ Aerogels Prepared by Ambient Pressure Drying with Ternary Azeotropes as Components of Pore Fluid. J. Non-Cryst. Solids.

[8] Wang J., Zhang Y., Wei Y., Zhang X. (2015). Fast and One-Pot Synthesis of Silica Aerogels via a Quasi-solvent-exchange-free Ambient Pressure Drying Process. Microporous Mesoporous Mater..

[9] Hayase G., Kanamori K., Maeno A., Kaji H., Nakanishi K. (2016). Dynamic Spring-back Behavior in Evaporative Drying of Polymethylsilsesquioxane Monolithic Gels for Low-density Transparent Thermal Superinsulators. J. Non-Cryst. Solids.

[10] Sarawade P.B., Kim J.-K., Hilonga A., Kim H.T. (2010). Preparation of Hydrophobic Mesoporous Silica Powder with a High Specific Surface Area by Surface Modification of a Wet-Gel Slurry and Spray-Drying. Powder Technol..

[11] Barnyakov A.Y., Barnyakov M.Y., Bahr J., Bellunato T., Beloborodov K.I., Bobrovnikov V.S., Buzykaev A.R., Calvi M., Danilyuk A.F., Djordjadze V. (2005). Development of Aerogel Cherenkov Detectors at Novosibirsk. Nucl. Instrum. Methods Phys. Res. Sect. A.

[12] Barnyakov A.Y., Barnyakov M.Y., Bobrovnikov V.S., Buzykaev A.R., Danilyuk A.F., Gulevich V.V., Kirillov V.L., Kononov S.A., Kravchenko E.A., Onuchin A.P. (2008). Focusing Aerogel RICH Optimization. Nucl. Instrum. Methods Phys. Res. Sect. A.

[13] Barnyakov A.Y., Barnyakov M.Y., Beloborodov K.I., Bobrovnikov V.S., Buzykaev A.R., Golubev V.B., Gulevich B.V., Danilyuk A.F., Kononov S.A., Kravchenko E.A. (2011). Status of Aerogel Production in Novosibirsk. Nucl. Instrum. Methods Phys. Res. Sect. A.

[14] Ishii Y., Adachi I., Kawai H., Saito Y., Sumiyoshi T., Tabata M., Yokogawa H. Development of Silica Aerogel with Refractive Index Gradient for RICH Radiator. Proceedings of the IEEE Nuclear Science Symposium Conference Record.

[15] Tabata M., Adachi I., Kawai H., Sumiyoshi T., Yokogawa H. (2012). Hydrophobic Silica Aerogel Production at KEK. Nucl. Instrum. Methods Phys. Res. Sect. A.

[16] Tabata M., Adachi I., Hatakeyama Y., Kawai H., Morita T., Sumiyoshi T. (2016). Large-area Silica Aerogel for Use as Cherenkov Radiators with High Refractive Index, Developed by Supercritical Carbon Dioxide Drying. J. Supercrit. Fluids.

[17] Tajiri K., Igarashi K., Nishio T. (1995). Effects of Supercritical Drying Media on Structure and Properties of Silica Aerogel. J. Non-Cryst. Solids.

[18] Laudise R., Johnson D. (1986). Supercritical Drying of Gels. J. Non-Cryst. Solids.

[19] Kocon L., Despetis F., Phalippou J. (1998). Ultralow Density Silica Aerogels by Alcohol Supercritical Drying. J. Non-Cryst. Solids.

[20] Stolarski M., Walendziewski J., Steininger M., Pniak B. (1999). Synthesis and Characteristic of Silica Aerogels. Appl. Catal. A Gen..

[21] Mahadik D.B., Lee Y.K., Chavan N.K., Mahadik S.A., Park H.-H. (2016). Monolithic and Shrinkage-free Hydrophobic Silica Aerogels via New Rapid Supercritical Extraction Process. J. Supercrit. Fluids.

[22] Kong Y., Shen X.-D., Cui S. (2014). Direct Synthesis of Anatase TiO_2_ Aerogel Resistant to High Temperature under Supercritical Ethanol. Mater. Lett..

[23] Kirkbir F., Murata H., Meyers D., Chaudhuri S.R. (1998). Drying of Large Monolithic Aerogels between Atmospheric and Supercrititcal Pressures. J. Sol-Gel Sci. Technol..

[24] Kirkbir F., Murata H., Meyers D., Chaudhuri S.R. (1998). Drying of Aerogels in Different Solvents between Atmospheric and Supercritical Pressures. J. Non-Cryst. Solids.

[25] Dowson M., Grogan M., Birks T., Harrison D., Craig S. (2012). Streamlined Life Cycle Assessment of Transparent Silica Aerogel Made by Supercritical Drying. Appl. Energy.

[26] Van Bommel M.J., de Haan A.B. (1995). Drying of Silica Aerogel with Supercritical Carbon Dioxide. J. Non-Cryst. Solids.

[27] Sanz-Moral L.M., Rueda M., Nieto A., Novak Z., Knez Ž., Martín Á. (2013). Gradual Hydrophobic Surface Functionalization of Dry Silica Aerogels by Reaction with Silane Precursors Dissolved in Supercritical Carbon Dioxide. J. Supercrit. Fluids.

[28] Pajonk G.M., Venkateswara Rao A., Sawant B.M., Parvathy N.N. (1997). Dependence of Monolithicity and Physical Properties of TMOS Silica Aerogels on Gel Aging and Drying Conditions. J. Non-Cryst. Solids.

[29] Griffin J.S., Mills D.H., Cleary M., Nelson R., Manno V.P., Hodes M. (2014). Continuous Extraction Rate Measurements during Supercritical CO_2_ Drying of Silica Alcogel. J. Supercrit. Fluids.

[30] Masmoudi Y., Rigacci A., Ilbizian P., Cauneau F., Achard P. (2006). Diffusion during the Supercritical Drying of Silica Gels. Dry. Technol..

[31] García-González C.A., Camino-Rey M.C., Alnaief M., Zetzl C., Smirnova I. (2012). Supercritical Drying of Aerogels Using CO_2_: Effect of Extraction Time on the End Material Textural Properties. J. Supercrit. Fluids.

[32] Quiño J., Ruehl M., Klima T., Ruiz F., Will S., Braeuer A. (2016). Supercritical Drying of Aerogel: In Situ Analysis of Concentration Profiles inside the Gel and Derivation of the Effective Binary Diffusion Coefficient Using Raman Spectroscopy. J. Supercrit. Fluids.

[33] Wang Y.-Y., Gao Y.-B., Sun Y.-H., Chen S.-Y. (1996). Effect of Preparation Parameters on the Texture of SiO_2_ Aerogels. Catal. Today.

[34] Özbakır Y., Erkey C. (2015). Experimental and Theoretical Investigation of Supercritical Drying of Silica Alcogels. J. Supercrit. Fluids.

[35] Sanz-Moral L.M., Rueda M., Mato R., Martín Á. (2014). View Cell Investigation of Silica Aerogels during Supercritical Drying: Analysis of Size Variation and Mass Transfer Mechanisms. J. Supercrit. Fluids.

[36] Rao A.V., Pajonk G.M., Bhagat S.D., Barboux P. (2004). Comparative Studies on the Surface Chemical Modification of Silica Aerogels Based on Various Organosilane Compounds of the Type R*_n_*SiX_4-*n*_. J. Non-Cryst. Solids.

[37] Venkateswara Rao A., Pajonk G.M., Haranath D., Wagh P.B. (1998). Effect of Sol-Gel Processing Parameters on Optical Properties of TMOS Silica Aerogels. J. Mater. Synth. Process..

[38] Strøm R.A., Masmoudi Y., Rigacci A., Petermann G., Gullberg L., Chevalier B., Einarsrud M.-A. (2007). Strengthening and Aging of Wet Silica Gels for Up-Scaling of Aerogel Preparation. J. Sol-Gel Sci. Technol..

[39] Omranpour H., Motahari S. (2013). Effects of Processing Conditions on Silica Aerogel during Aging: Role of Solvent, Time and Temperature. J. Non-Cryst. Solids.

[40] Rigacci A., Einarsrud M.-A., Nilsen E., Pirard R., Ehrburger-Dolle F., Chevalier B. (2004). Improvement of the Silica Aerogel Strengthening Process for Scaling-up Monolithic Tile Production. J. Non-Cryst. Solids.

[41] He F., Zhao H., Qu X., Zhang C., Qiu W. (2009). Modified Aging Process for Silica Aerogel. J. Mater. Process. Technol..

