# Supplementary material for: A Continuous Extraction and Pumpless Supercritical CO2 Drying System for Laboratory-Scale Aerogel Production
Source: Gels. 2016 Oct 1;2(4):26. doi: 10.3390/gels2040026 (PMC6318629; doi:10.3390/gels2040026)
Supplement: Supplementary file 1 [file gels-02-00026-s001.zip › gels-02-00026-s001.docx]

**Supplementary Materials: A Continuous Extraction and Pumpless Supercritical CO_2_ Drying System for Laboratory-Scale Aerogel Production**

István Lázár and István Fábián


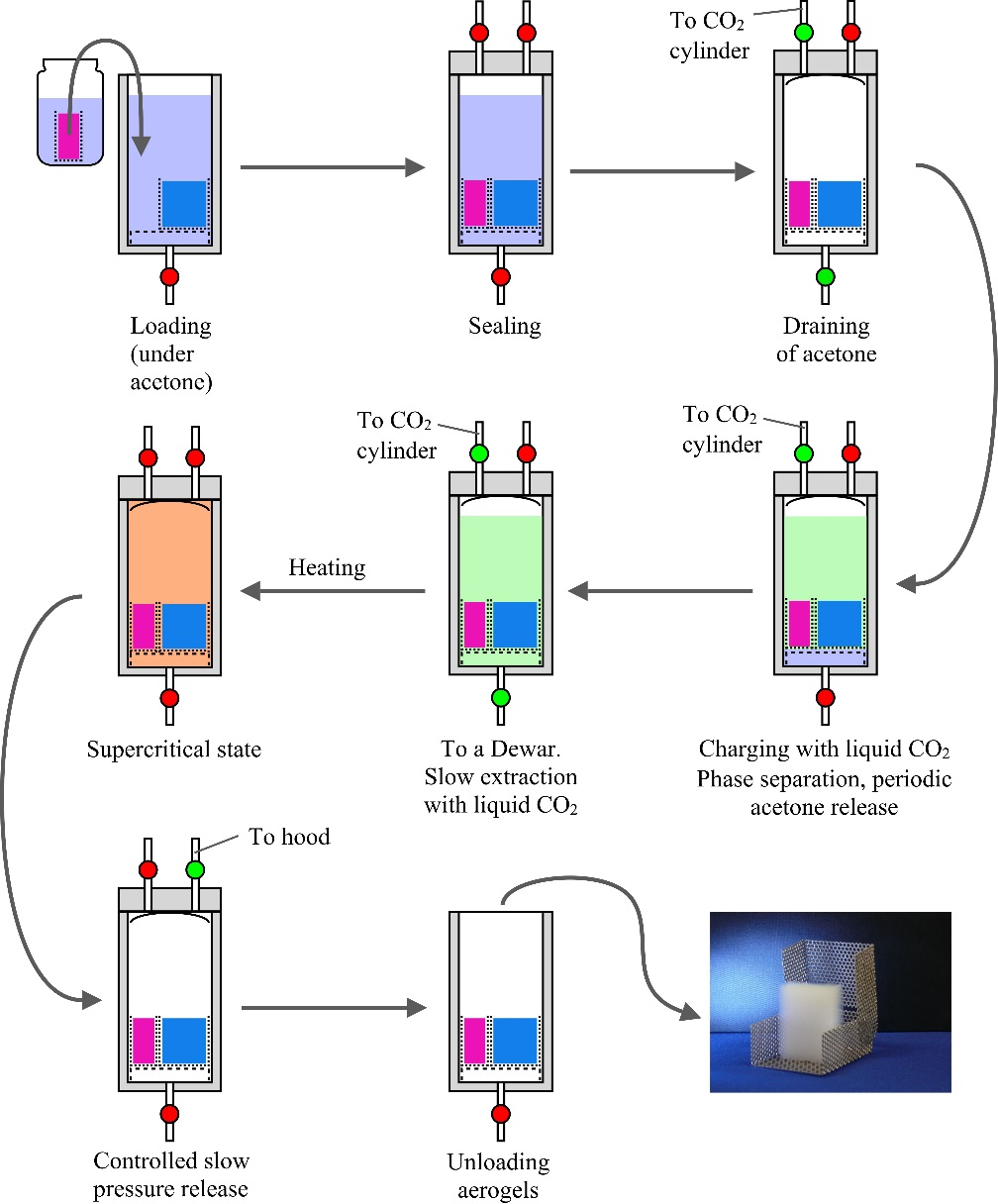


**Figure S1.** Visualization of important operation steps and valve settings of liquid carbon dioxide extraction and MT supercritical drying of aerogels. Red circles: valve closed, green circles: valve open.


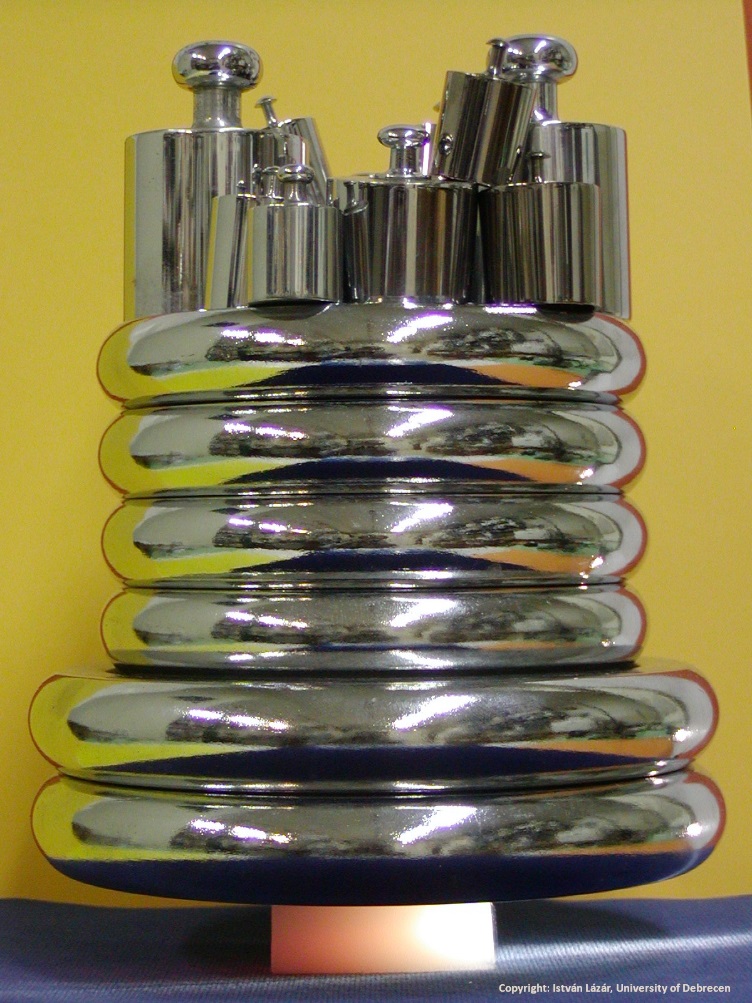


**Figure S2.** Acid-base catalysed aerogel sample holding 10 kg weight without cracking.


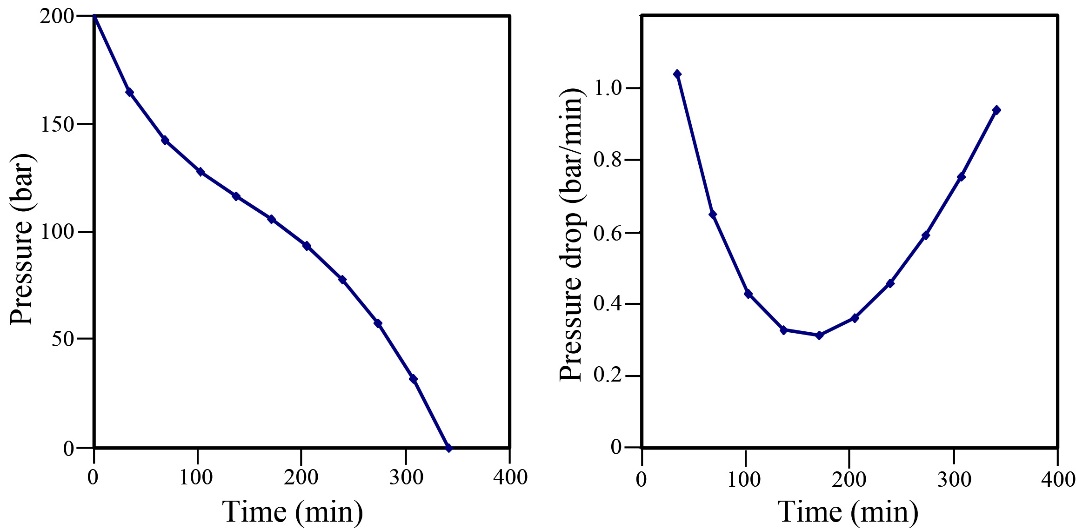


**Figure S3.** Change of pressure (panel **left**) and pressure drop (panel **right**) as a function of time, calculated by the van der Waals equation for modeling a constant volumetric rate of 1.3 L/min (STP) decompression process of a 1.5 L dryer, which was filled with 1 L of liquid carbon dioxide at 291 K temperature and 5.4 MPa pressure, and heated to 353 K before decompression.
